# Supplementary material for: Illuminating phenotypic drug responses of sarcoma cells to kinase inhibitors by phosphoproteomics
Source: Mol Syst Biol. 2023 Dec 18;20(1):28–55. doi: 10.1038/s44320-023-00004-7 (PMC10883282; doi:10.1038/s44320-023-00004-7)
Supplement: Supplementary file 1 — Appendix Table S1-S2, Appendix Figure S1-S9 [file 44320_2023_4_MOESM1_ESM.pdf]

Table of Content

Appendix Table S1. Commercially or publicly available cell lines during cell line selection. 2
Appendix Table S2. EC50 values of Staurosporine in sarcoma lines. 3

Appendix Figure S1. Correlation of EC50 values (nM) of effective drugs across sarcoma lines between previous study and this study. 5
Appendix Figure S2. Correlation of intensity of the quantified proteins between CCLE proteomics analysis and this study. 6
Appendix Figure S3. Reproducibility of proteomics analysis in biological triplicate. 7
Appendix Figure S4. Reproducibility of phosphoproteomics analysis in biological triplicate. 8
Appendix Figure S5. Correlation of the intensity several G2/M markers with the response of mTOR inhibitor Ridaforolimus. 9
Appendix Figure S6. Clustering plot of correlations between drug responses among sarcoma cells. 10
Appendix Figure S7. Localization probability of phosphorylation sites. 11
Appendix Figure S8. Boxplot of baseline proteomes and phosphoproteomes before and after median-centric normalization. 12
Appendix Figure S9. Reproducibility of elastic net regression in Cobimetinib and Infigratinib. 13

Appendix Table S1. Commercially or publicly available cell lines during cell line selection.

CELL LINE	SUBTYPES
**A-204**	Rhabdoid tumor
**A-673**	Ewing Sarcoma
**G-401**	Rhabdoid Tumor
**HOS**	Osteosarcoma
**HS 706.T**	Giant Cell Sarcoma
**HS 729**	Rhabdomyosarcoma
**HS 913.T**	Fibrosarcoma
**HSSY-II**	Synovial sarcoma
**HT-1080**	Fibrosarcoma
**KHOS NP**	Osteosarcoma
**KHOS-240S**	Osteosarcoma
**KHOS-312H**	Osteosarcoma
**MES-SA**	Uterine Sarcoma
**RD**	Rhabdomyosarcoma
**RD-ES**	Ewing Sarcoma
**SAOS-2**	Osteosarcoma
**SJSA-1**	Osteosarcoma
**SK-ES-1**	Ewing Sarcoma
**SK-LMS-1**	Leiomyosarcoma
**SK-UT-1**	Leiomyosarcoma
**SK-UT-1B**	Leiomyosarcoma
**SW 1353**	Chondrosarcoma
**SW 684**	Fibrosarcoma
**SW 982**	Atypical Synovial sarcoma
**SYO-1**	Synovial sarcoma
**TC-71**	Ewing Sarcoma
**U-2 OS**	Osteosarcoma
**VA-ES-BJ**	Epithelioid Sarcoma

Appendix Table S2. EC50 values of Staurosporine in sarcoma lines.

CELL LINE	MEAN EC50 (µM)	STDEV*
**RD-ES**	0.033	0.0016
**MES-SA**	0.028	0.0027
**SW684**	0.027	0.0038
**SK-ES-1**	0.024	0.0010
**HS729**	0.023	0.0073
**SK-LMS-1**	0.013	0.0029
**KHOS-NP**	0.012	0.0020
**VA-ES-BJ**	0.007	0.0006
**HT1080**	0.007	0.0005
**G401**	0.006	0.0011
**RD**	0.006	0.0006
**A204**	0.005	0.0012
**SYO-1**	0.003	0.0001
**KHOS-240S**	0.003	0.0002
**SW872**	0.003	0.0003
**SW1353**	0.003	0.0001
**SW982**	0.001	0.0001

*Standard deviation from triplicate viability assays.

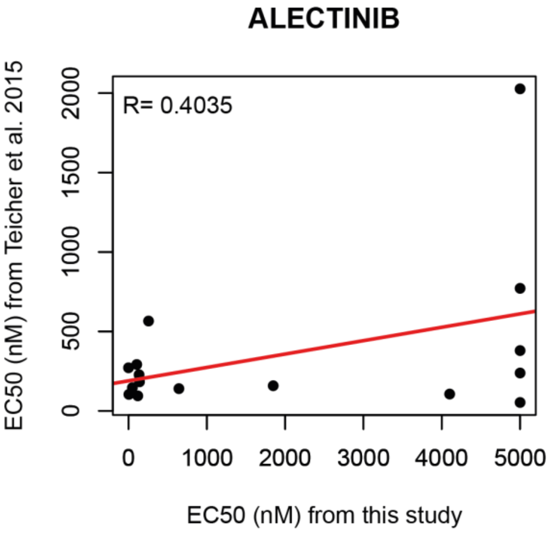
ALECTINIB
R= 0.4035
EC50 (nM) from Teicher et al. 2015
0 500 1000 1500 2000
0 1000 2000 3000 4000 5000
EC50 (nM) from this study

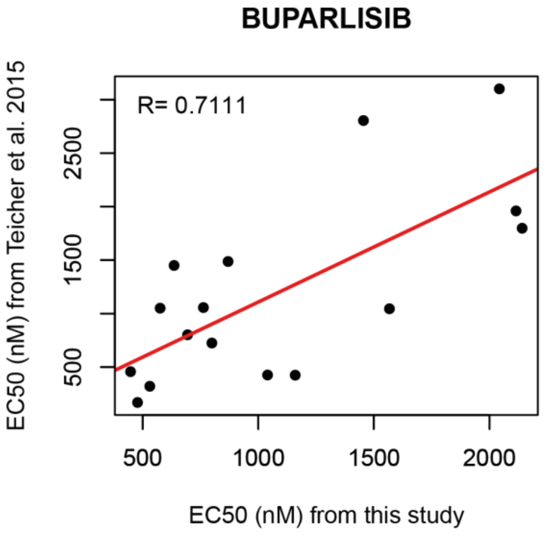
BUPARLISIB
R= 0.7111
EC50 (nM) from Teicher et al. 2015
500 1500 2500
500 1000 1500 2000
EC50 (nM) from this study

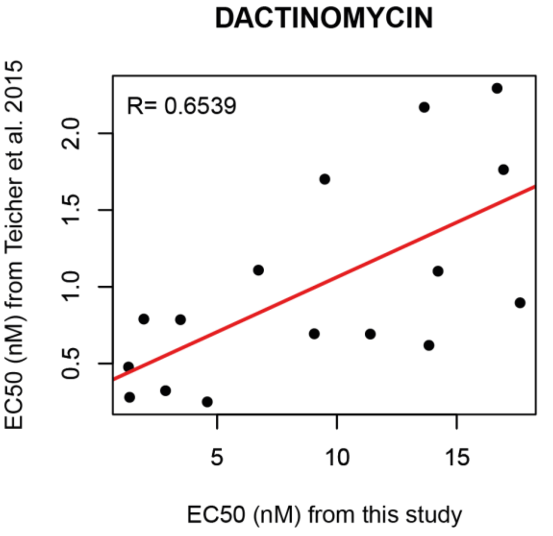
DACTINOMYCIN
R= 0.6539
EC50 (nM) from Teicher et al. 2015
0.5 1.0 1.5 2.0
5 10 15
EC50 (nM) from this study

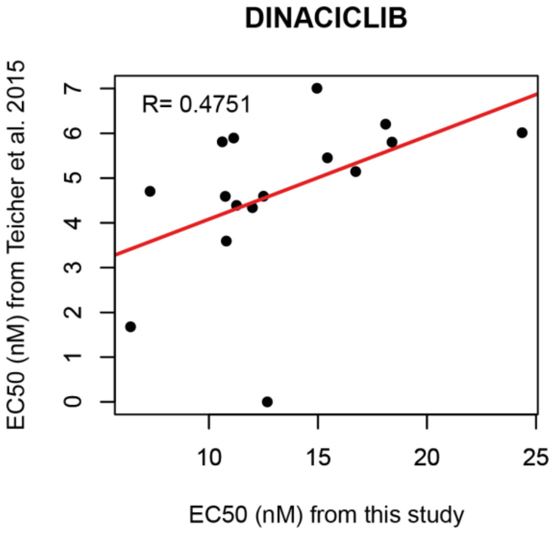
DINACICLIB
R= 0.4751
EC50 (nM) from Teicher et al. 2015
0 1 2 3 4 5 6 7
10 15 20 25
EC50 (nM) from this study

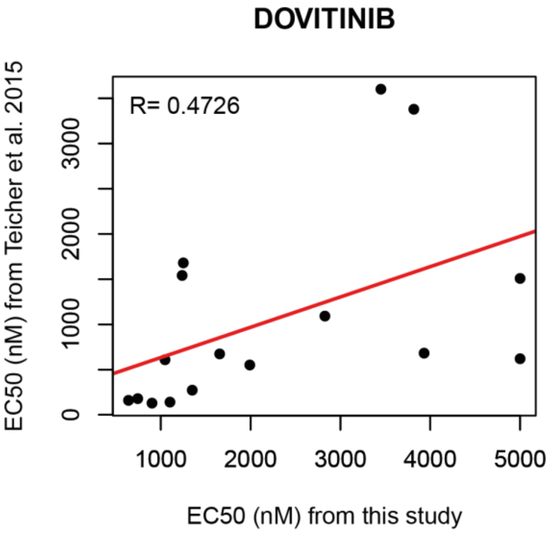
DOVITINIB
R= 0.4726
EC50 (nM) from Teicher et al. 2015
0 1000 2000 3000
1000 2000 3000 4000 5000
EC50 (nM) from this study

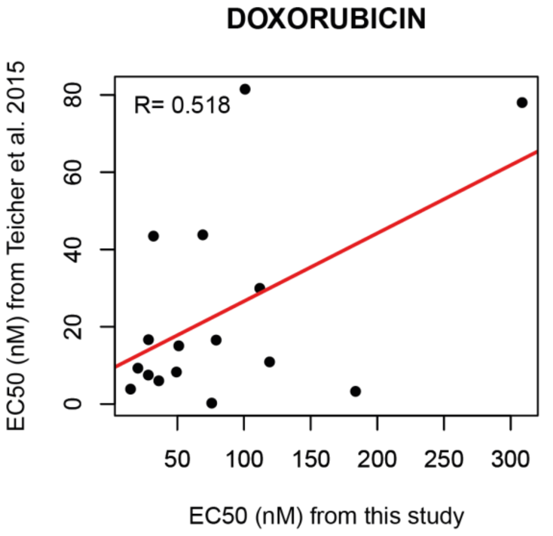
DOXORUBICIN
R= 0.518
EC50 (nM) from Teicher et al. 2015
0 20 40 60 80
50 100 150 200 250 300
EC50 (nM) from this study

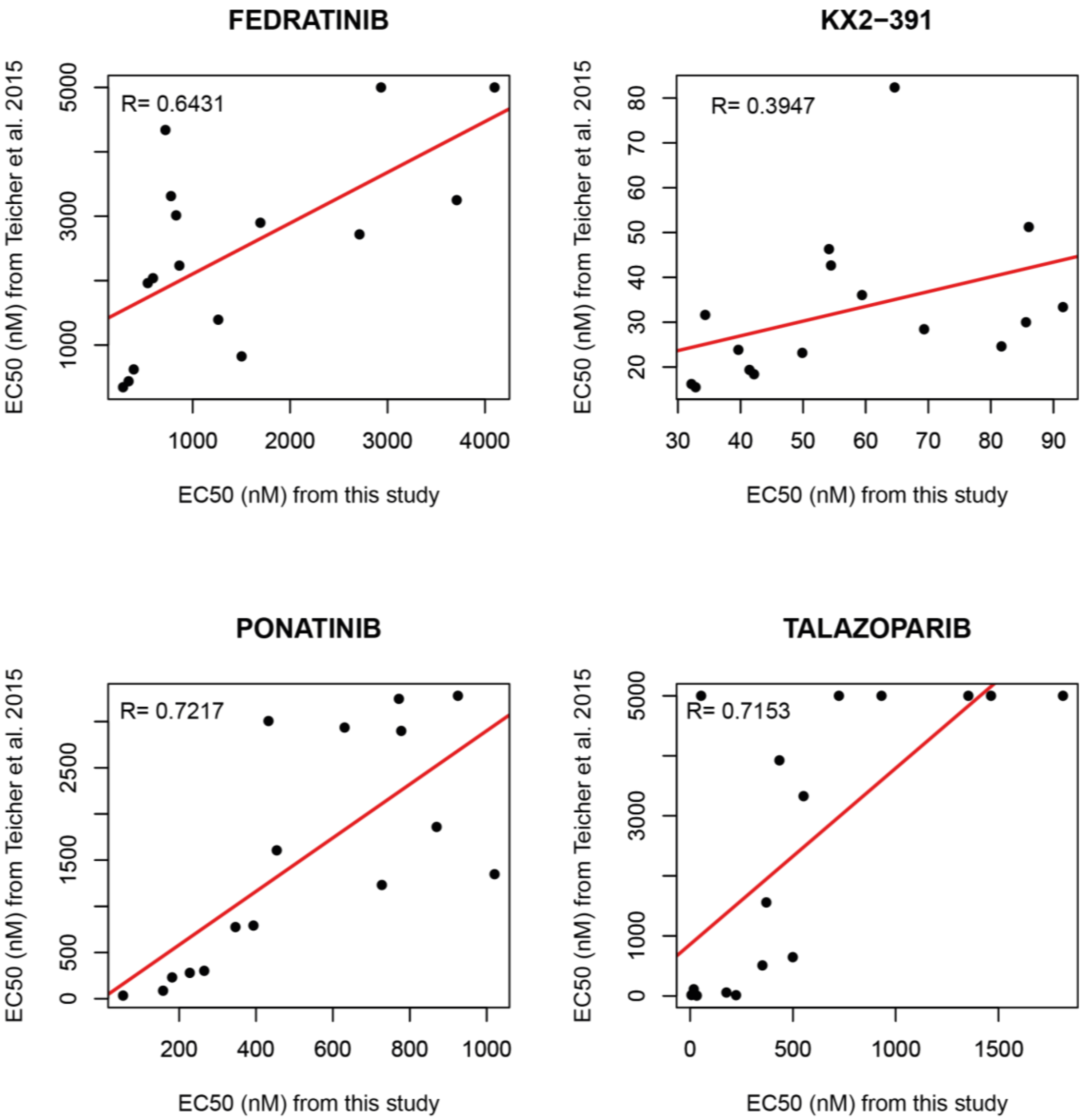

Appendix Figure S1. Correlation of EC50 values (nM) of effective drugs across sarcoma lines between previous study and this study. The drug screen dataset of 16 sarcoma lines was downloaded from precious study (Teicher *et al*, 2015) where 63 cell lines were screened with 445 compounds. In total, sixteen cell lines and 55 compound (52 KIs and 3 non-KIs) were shared with our screen library. Ten drugs shown here are the drugs with EC50 between 1 to 5000 nM across 16 cell lines in at least one of datasets. To make the plot clear, EC50 values > 5000 nM were plotted as 5000 nM.

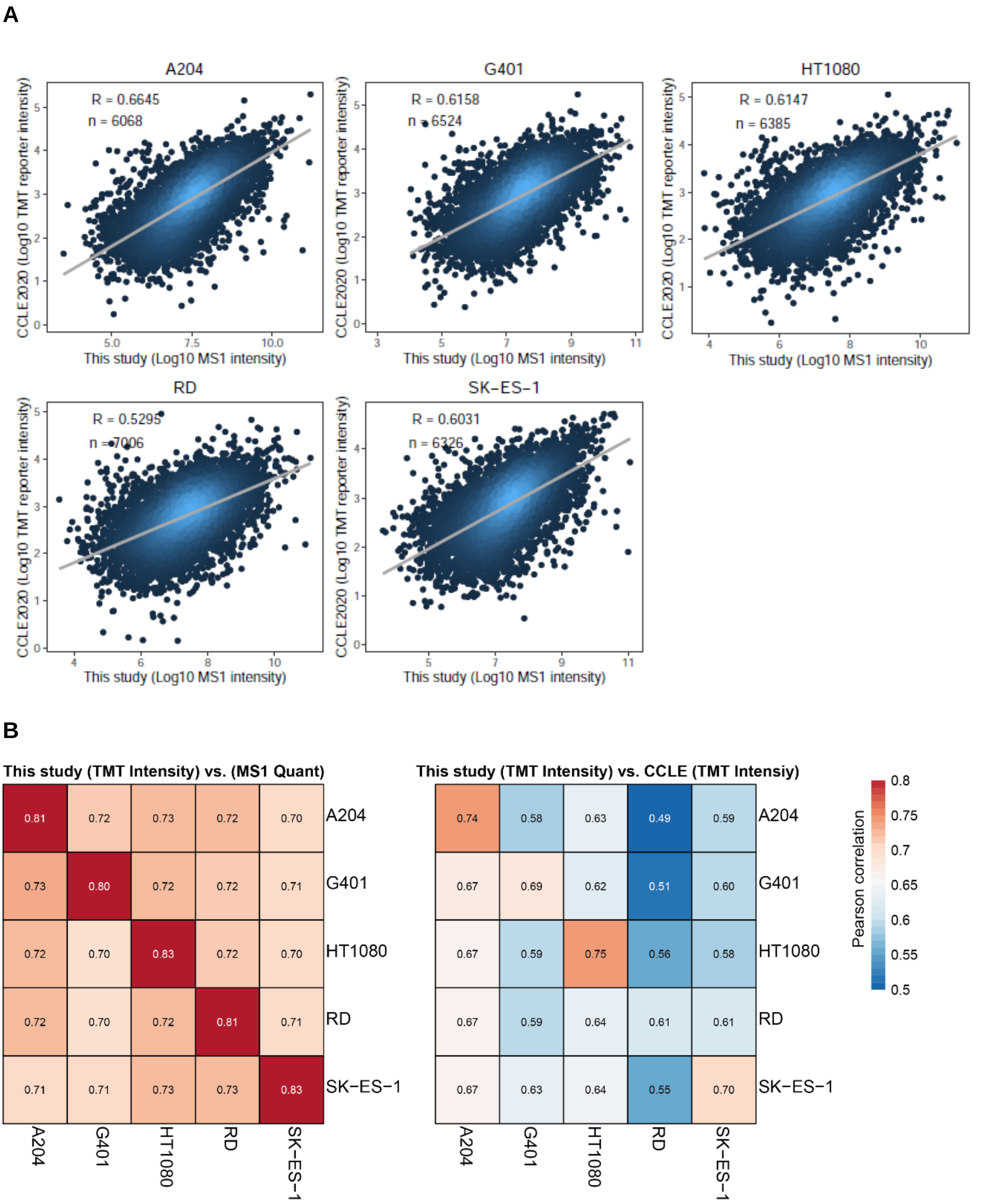

Appendix Figure S2. Correlation of intensity of the quantified proteins between CCLE proteomics analysis and this study. The proteomics dataset of CCLE 2020 was downloaded from precious study (Nusinow et al, 2020) where 375 cell lines from diverse lineages were multiplexed for proteomics analysis (TMT labelled). A. Five cell lines were shared between the two studies. The Pearson correlation and numbers of shared proteins were labelled on the plot. B. Comparison of correlations between matched and unmatched cell lines in our own datasets with MS1 quantification and TMT reporter intensity (left), as well as the datasets obtained from CCLE with TMT reporter intensity (right).

A

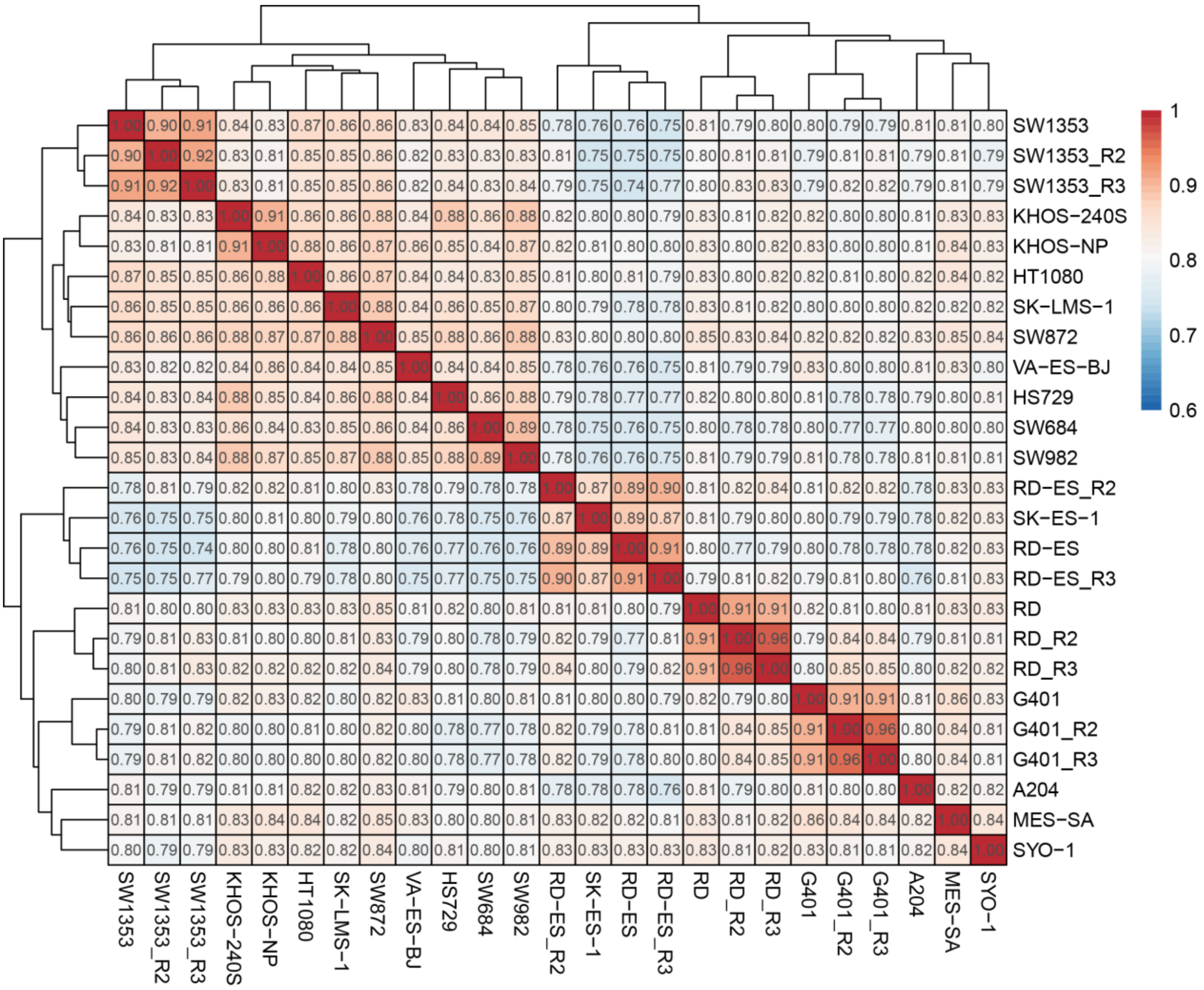

B

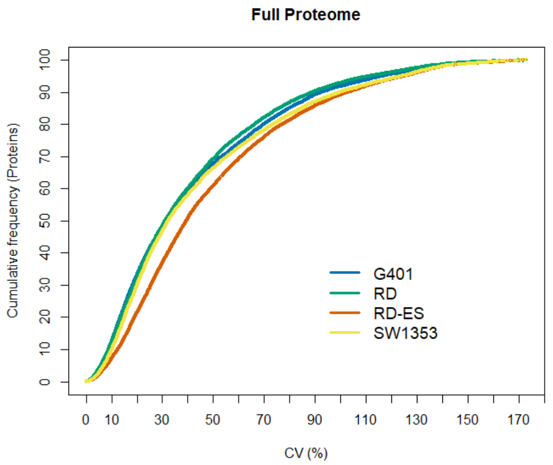

Appendix Figure S3. Reproducibility of proteomics analysis in biological triplicate. A. Unsupervised hierarchical clustering of 17 cell lines with 8 biological replicates cell lines. Pearson correlation was shown. R2, R3: biological replicates. B. Ranked protein coefficients of variation (CVs) of four triplicate cell lines.

A

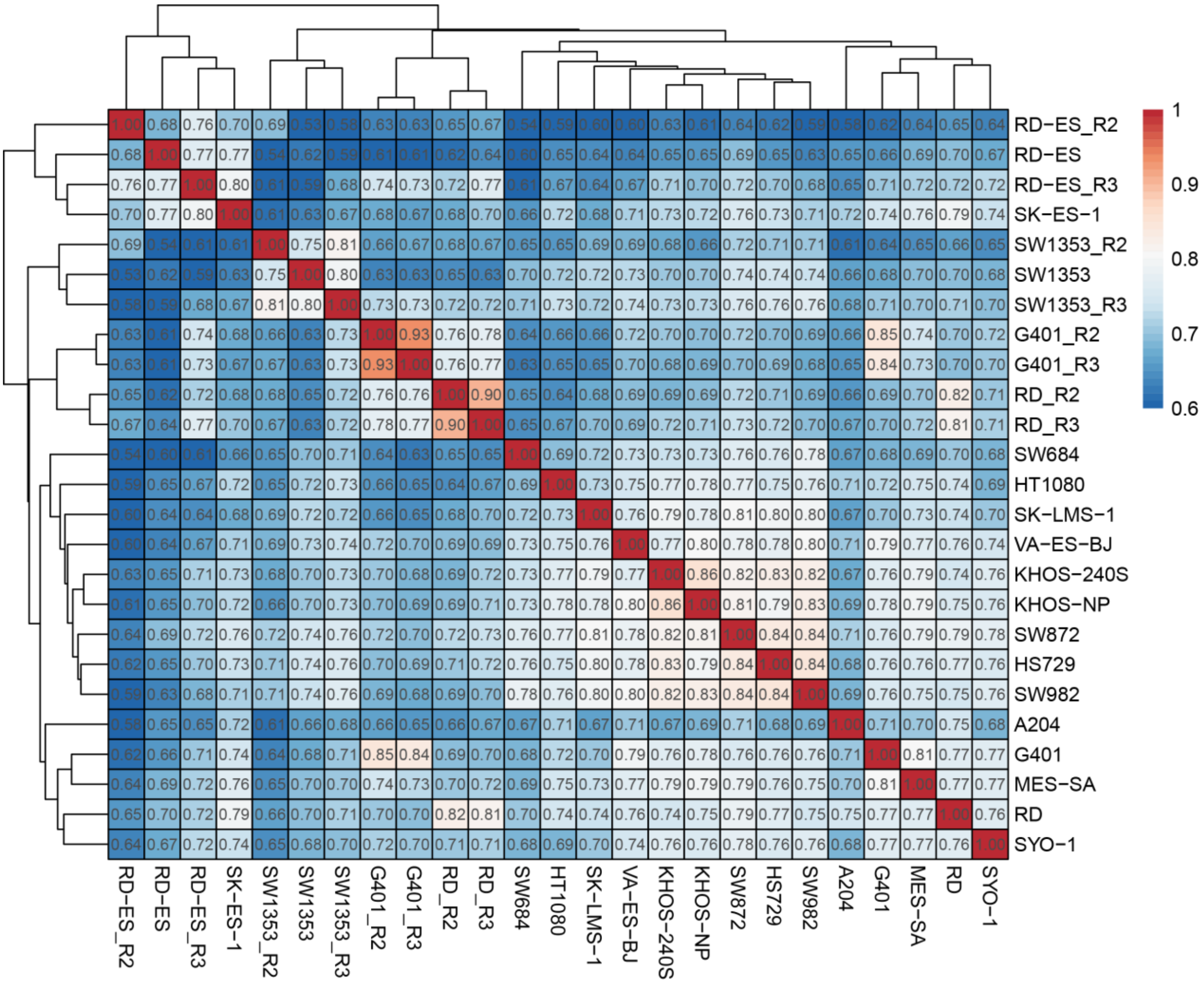

B

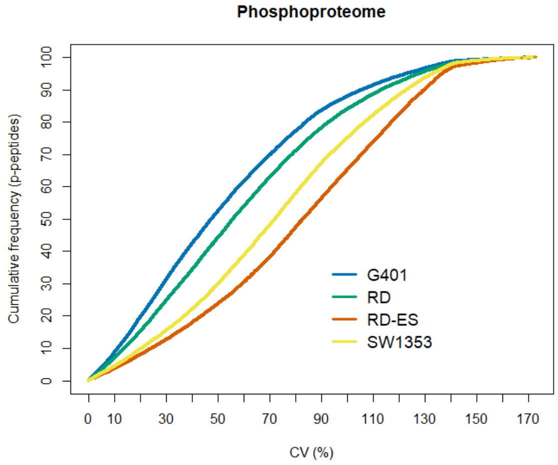

Appendix Figure S4. Reproducibility of phosphoproteomics analysis in biological triplicate. A. Unsupervised hierarchical clustering of 17 cell lines with eight biological replicates cell lines on phosphoprotein level. Pearson correlation was shown. R2, R3: biological replicates. B. Ranked phosphopeptides coefficients of variation (CVs) of four triplicate cell lines.

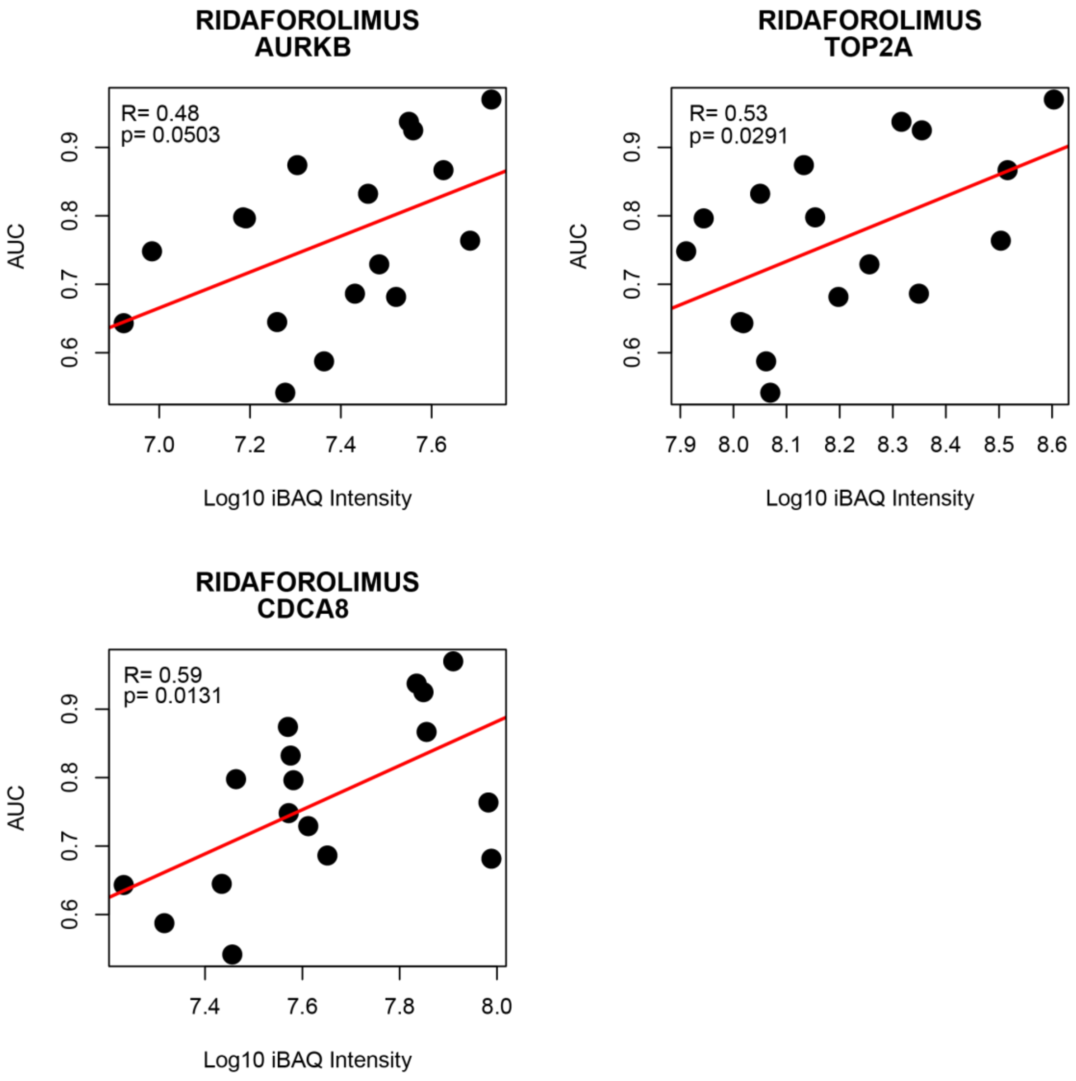

Appendix Figure S5. Correlation of the intensity several G2/M markers with the response of mTOR inhibitor Ridaforolimus.

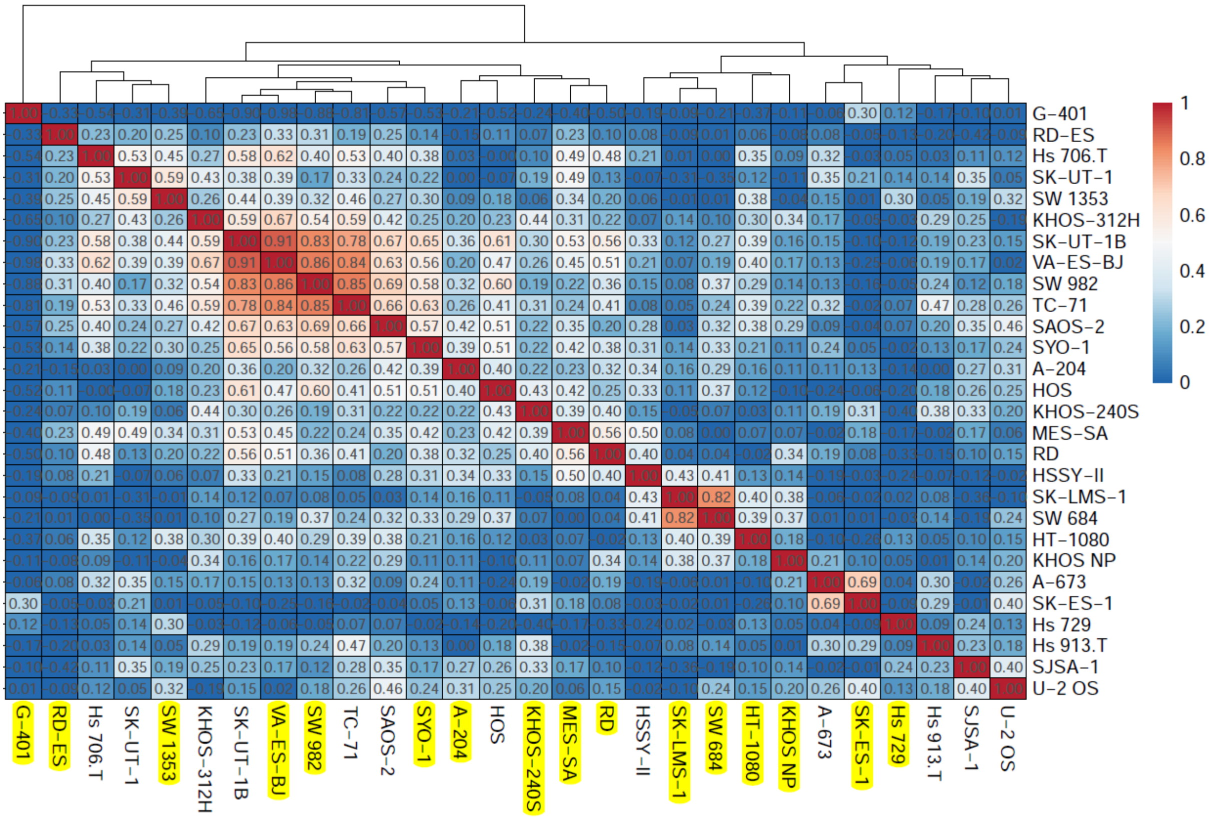

Appendix Figure S6. Clustering plot of correlations between drug responses among sarcoma cells. The Pearson correlation was calculated according to prior screen dataset (Teicher *et al.*, 2015). The cell lines chosen in this study were marked in yellow.

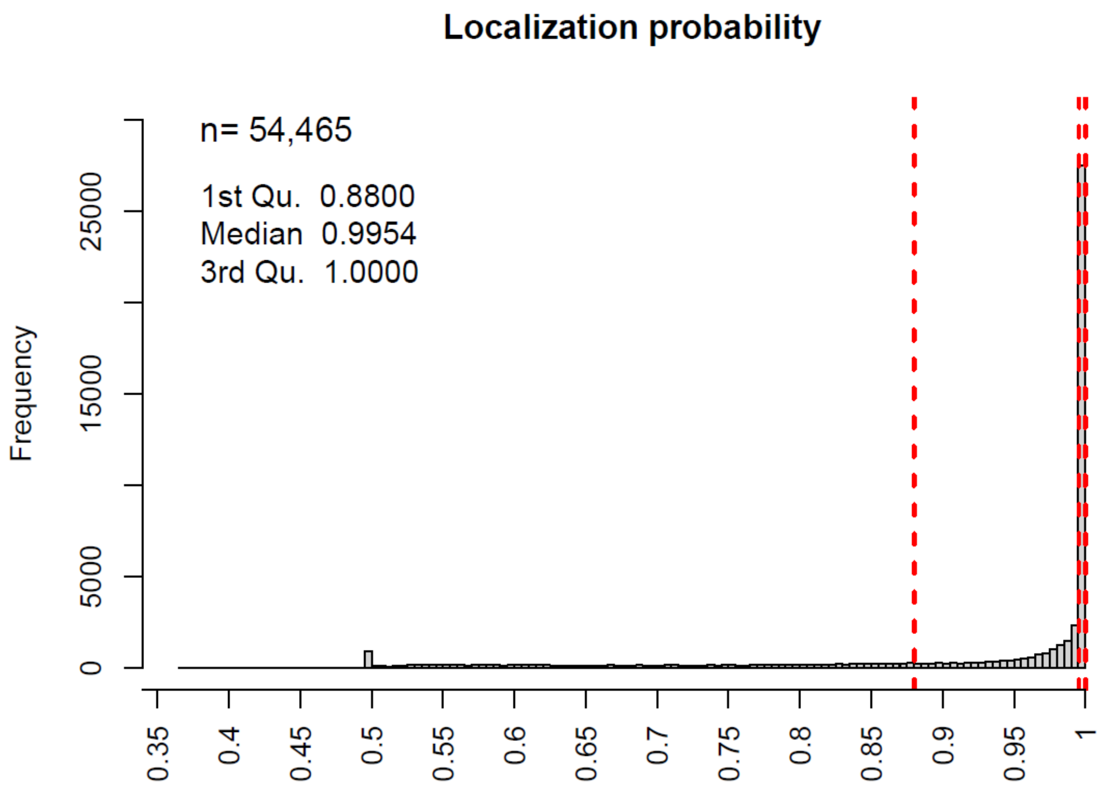

Appendix Figure S7. Localization probability of phosphorylation sites.

A Full Proteomes

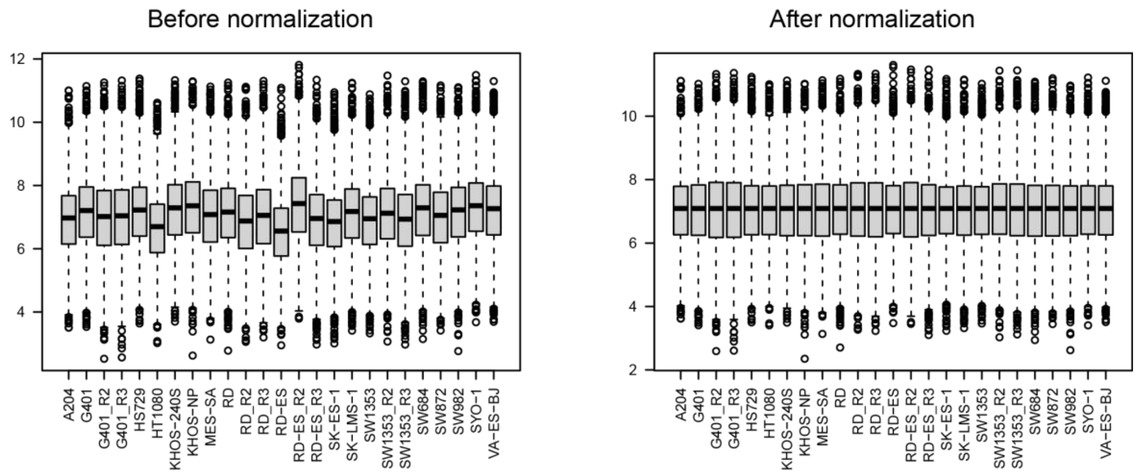

B Phosphoproteomes

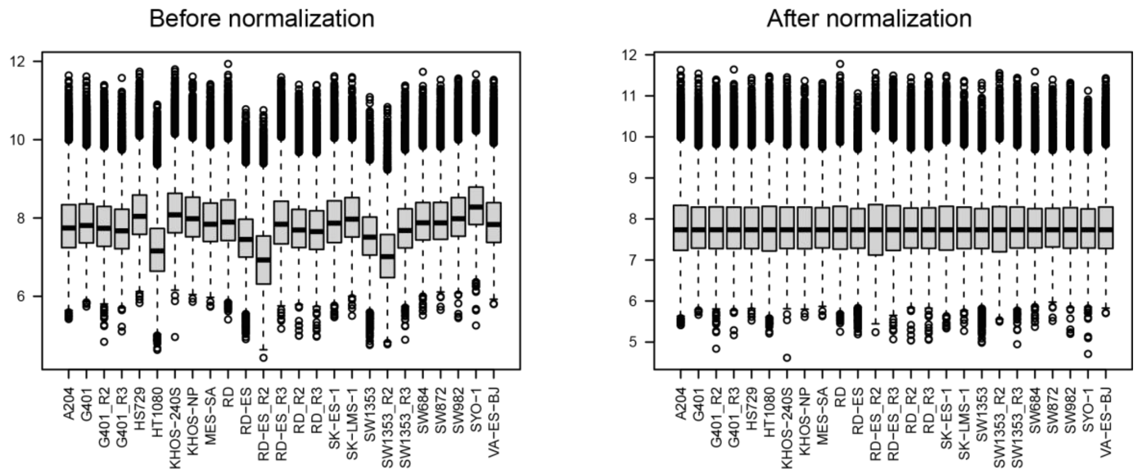

Appendix Figure S8. Boxplot of baseline proteomes and phosphoproteomes before and after median-centric normalization. A. Y-axis indicated log10-transformed iBAQ intensity. B. Y-axis indicated log10-transformed MS1 intensity. The central band represents the median, while the hinges denote the first and third quartiles with whiskers extending up to 1.5 times the interquartile range (IQR).

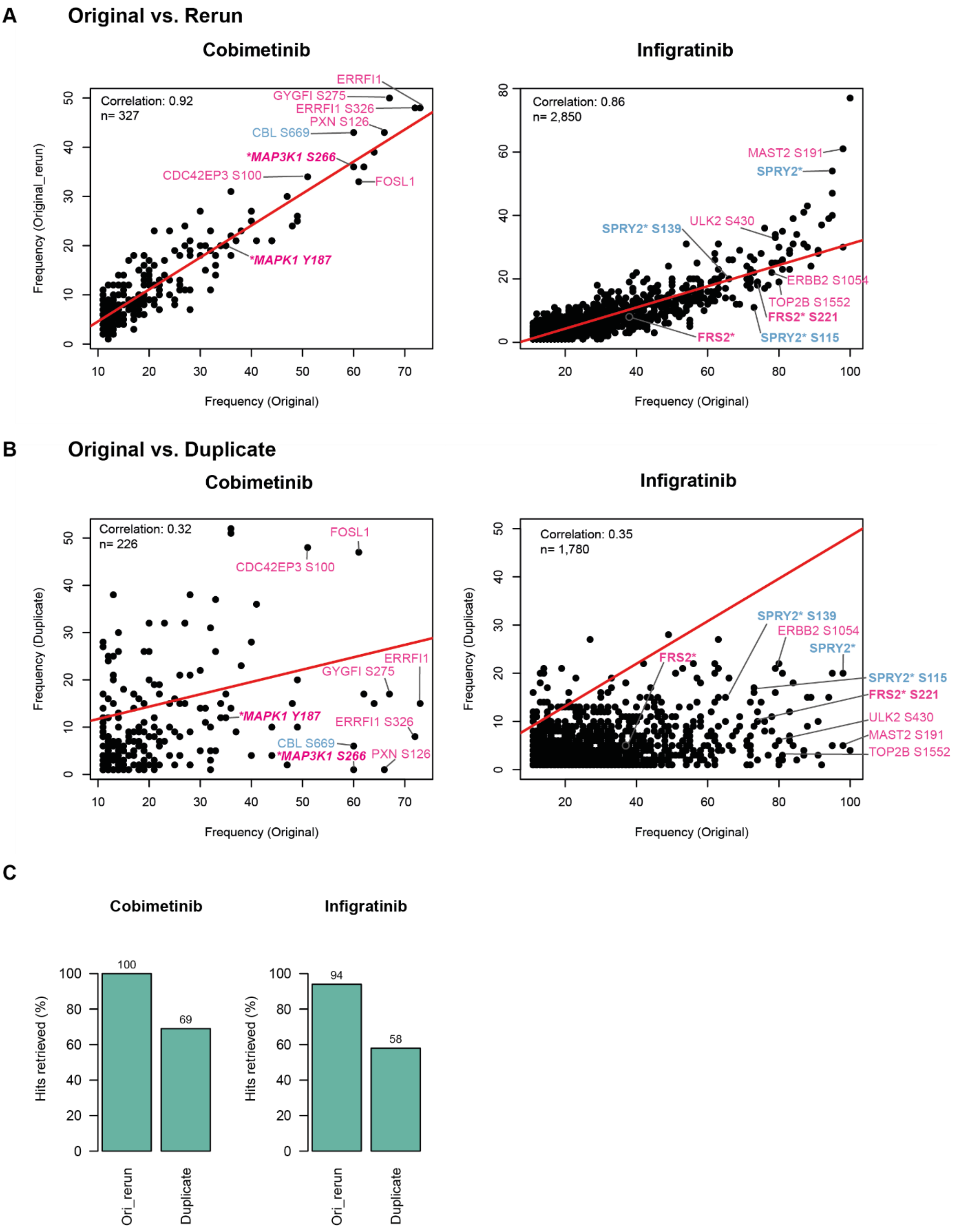

Appendix Figure S9. Reproducibility of elastic net regression in Cobimetinib and Infigratinib. A. The correlation of the frequencies of identified proteins and p-sites in original and rerun elastic net regression. B. The correlation of the frequencies of identified proteins and p-sites in original and duplicate datasets. The labels show the chosen example in main Figure 5. C. The percentage of retrieved proteins and p-sites compared to original analysis.

References

Nusinow DP, Szpyt J, Ghandi M, Rose CM, McDonald ER, 3rd, Kalocsay M, Jane-Valbuena J, Gelfand E, Schweppe DK, Jedrychowski M *et al* (2020) Quantitative Proteomics of the Cancer Cell Line Encyclopedia. *Cell* 180: 387-402 e316

Teicher BA, Polley E, Kunkel M, Evans D, Silvers T, Delosh R, Laudeman J, Ogle C, Reinhart R, Selby M *et al* (2015) Sarcoma Cell Line Screen of Oncology Drugs and Investigational Agents Identifies Patterns Associated with Gene and microRNA Expression. *Mol Cancer Ther* 14: 2452-2462
