# Supplementary material for: Illuminating phenotypic drug responses of sarcoma cells to kinase inhibitors by phosphoproteomics
Source: Mol Syst Biol. 2023 Dec 18;20(1):28–55. doi: 10.1038/s44320-023-00004-7 (PMC10883282; doi:10.1038/s44320-023-00004-7)
Supplement: Supplementary file 15 — Expanded View Figures [file 44320_2023_4_MOESM15_ESM.pdf]

Expanded View Figures

Figure EV1. Summary of data quality.

(**A**) Distributions of coefficient of variation (CV) of AUC (area under the curve) values determined from cell viability assays ($n = 3$) in response to 150 cancer drugs (each circle represents one drug). The central band represents the median, while the hinges denote the first and third quartiles with whiskers extending up to 1.5 times the interquartile range (IQR). (**B**) The number of quantified protein groups/peptides (left) and p-sites/phosphorylated peptides (right). (**C**) Unsupervised hierarchical clustering of biological triplicates of four cell lines on protein (left) and phosphoprotein (right) level. Pearson correlation was shown. R2, R3: biological replicates.

© The Author(s)

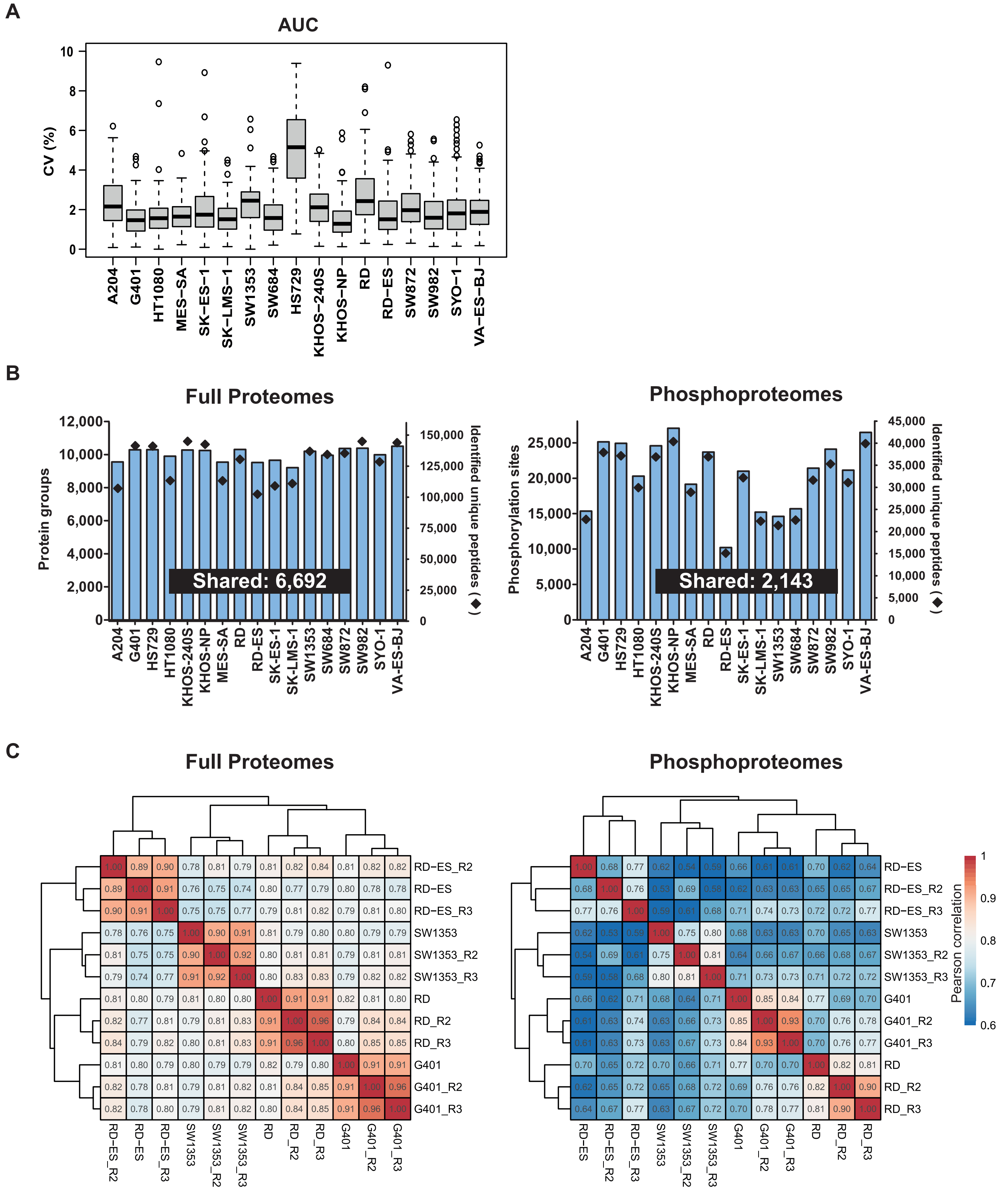
A
AUC
CV (%)
A204
G401
HT1080
MES−SA
SK−ES−1
SK−LMS−1
SW1353
SW684
HS729
KHOS−240S
KHOS−NP
RD
RD−ES
SW872
SW982
SYO−1
VA−ES−BJ
B
Full Proteomes
Protein groups
Identified unique peptides (◆)
Shared: 6,692
Phosphoproteomes
Phosphorylation sites
Identified unique peptides (◆)
Shared: 2,143
C
Full Proteomes
Phosphoproteomes
Pearson correlation
RD−ES_R2
RD−ES
RD−ES_R3
SW1353
SW1353_R2
SW1353_R3
RD
RD_R2
RD_R3
G401
G401_R2
G401_R3

 © The Author(s)

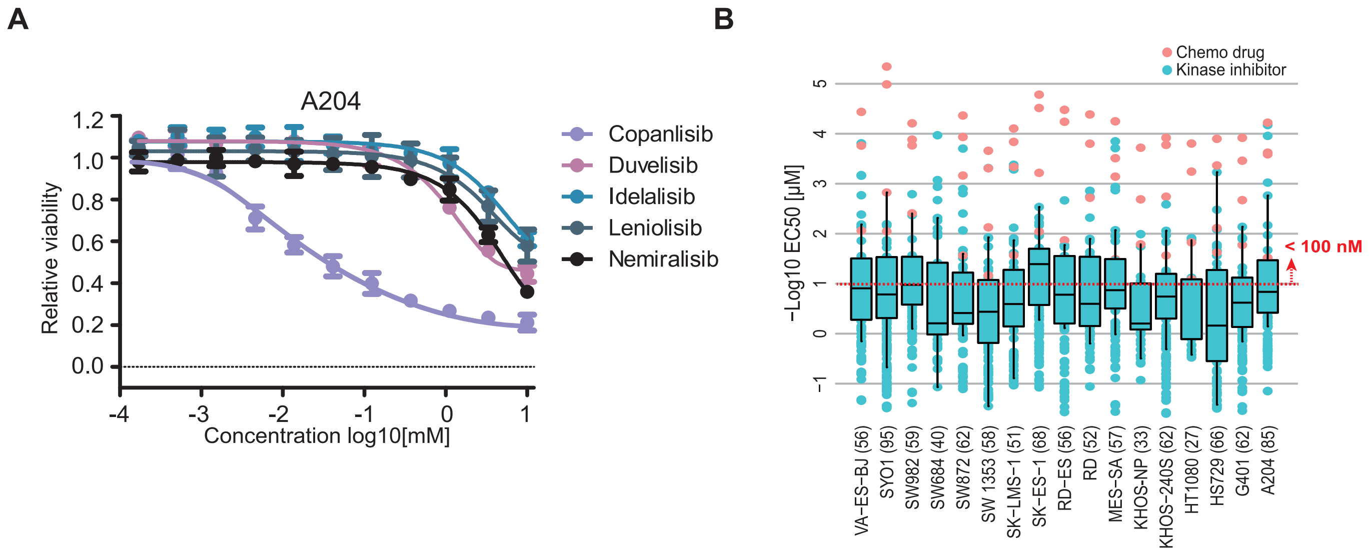

Figure EV2. Summary of the phenotypic dose–response characteristics of all cell lines and all drugs.

(**A**) Viability curves of five selected PI3K inhibitors in A204 cells. Measurements were in technical triplicates and the error bars were shown in SD. (**B**) Distribution of calculated EC50 values for all cell lines and all drugs. Numbers in brackets after each cell line name indicate the number of drugs that show effects larger than AUC>0.9 and have relative effect sizes of >50%. The red dotted line marks an EC50 value of 100 nM. The central band represents the median, while the hinges denote the first and third quartiles with whiskers extending up to 1.5 times the IQR.

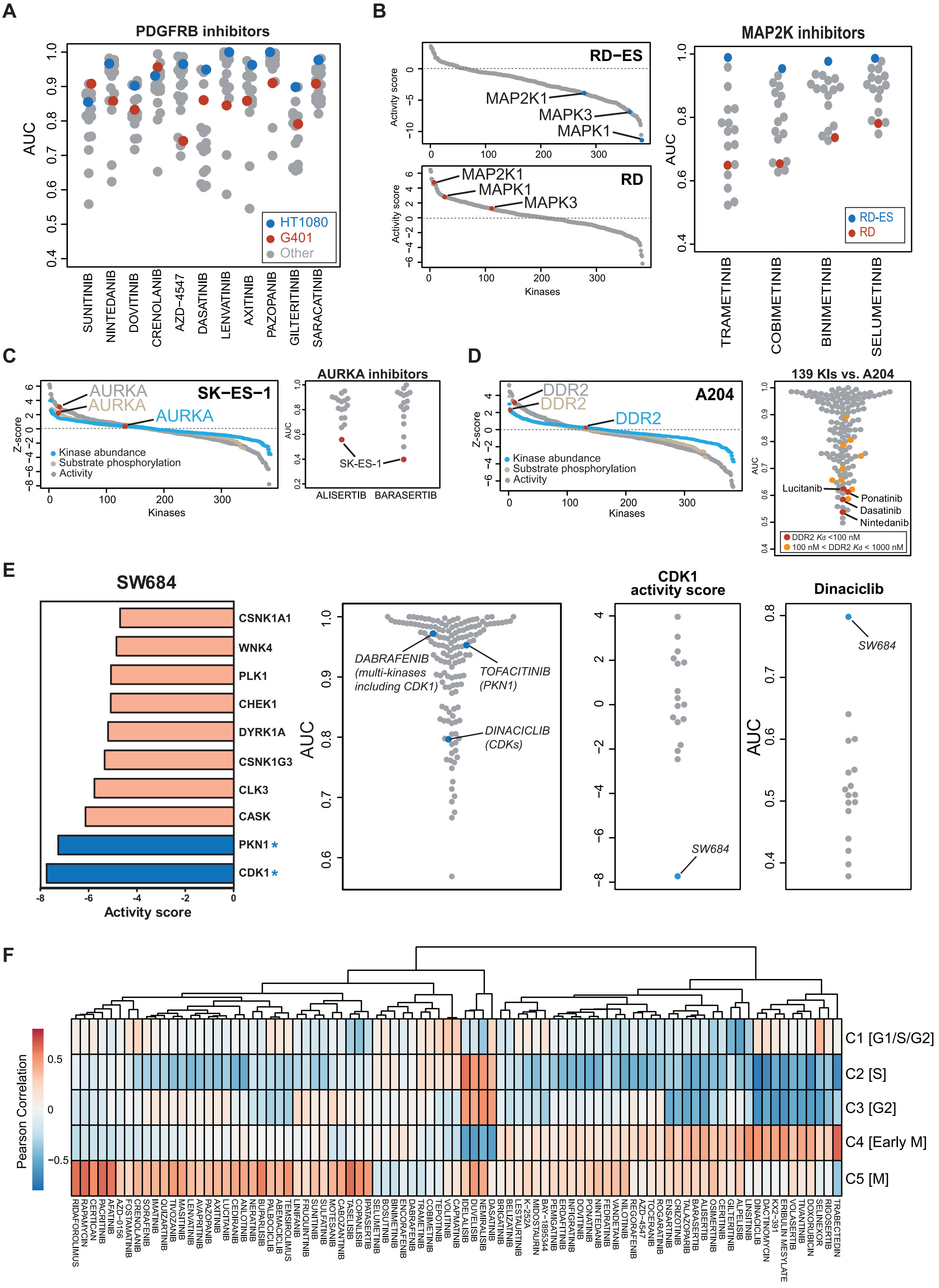
A
PDGFRB inhibitors
AUC
HT1080
G401
Other
SUNITINIB
NINTEDANIB
DOVITINIB
CRENOLANIB
AZD−4547
DASATINIB
LENVATINIB
AXITINIB
PAZOPANIB
GILTERITINIB
SARACATINIB
B
RD−ES
RD
MAP2K1
MAPK3
MAPK1
Activity score
Kinases
MAP2K inhibitors
AUC
RD-ES
RD
TRAMETINIB
COBIMETINIB
BINIMETINIB
SELUMETINIB
C
SK−ES−1
AURKA
Kinase abundance
Substrate phosphorylation
Activity
Z-score
Kinases
AURKA inhibitors
AUC
SK-ES-1
ALISERTIB
BARASERTIB
D
A204
DDR2
Kinase abundance
Substrate phosphorylation
Activity
Z-score
Kinases
139 KIs vs. A204
AUC
Lucitanib
Ponatinib
Dasatinib
Nintedanib
DDR2 Kd <100 nM
100 nM < DDR2 Kd < 1000 nM
E
SW684
CSNK1A1
WNK4
PLK1
CHEK1
DYRK1A
CSNK1G3
CLK3
CASK
PKN1 *
CDK1 *
Activity score
AUC
DABRAFENIB (multi-kinases including CDK1)
TOFACITINIB (PKN1)
DINACICLIB (CDKs)
CDK1 activity score
SW684
Dinaciclib
AUC
SW684
F
Pearson Correlation
C1 [G1/S/G2]
C2 [S]
C3 [G2]
C4 [Early M]
C5 [M]

© The Author(s)

Figure EV3. Integration of phenotypic drug response and proteomic activity scores.

(**A**) Phenotypic drug response of two sarcoma lines towards 11 PDGFRB inhibitors. (**B**) Distribution of activity scores of proteins in RD-ES (top panel) and RD cells (bottom panel) showing that MAP2K1 and its downstream substrates MAPK1 and MAPK3 have different activities in the two cell lines. This translates into differences in phenotypic response of the two cell lines to MAP2K inhibitors (right panel). (**C**) Ranked list of kinases either considering kinase abundance, substrate phosphorylation abundance or computed activity in SK-ES-1 cells (left panel) showing that AURKA phosphorylation and activity is substantially higher than protein abundance. This translates into a strong response of the cell lines to AURKA inhibitors (right panel). (**D**) Same as panel (**C**) but for DDR2 in A204 cells. (**E**) Left panel: list of kinases ranked by activity score in SW684 cells. The asterisk denotes a kinase that is a target of the kinase inhibitor. Middle panel: relative sensitivity of SW684 cells to all drugs in the screen. Right 2 panels: CDK1 activity score and drug responses toward Dinaciclib among 17 sarcoma lines. (**F**) The heatmap summarises the Pearson correlations of proportions of cell cycle clusters (from Kelly et al) and drug responses from 87 drugs (AUC <0.8).

© The Author(s)

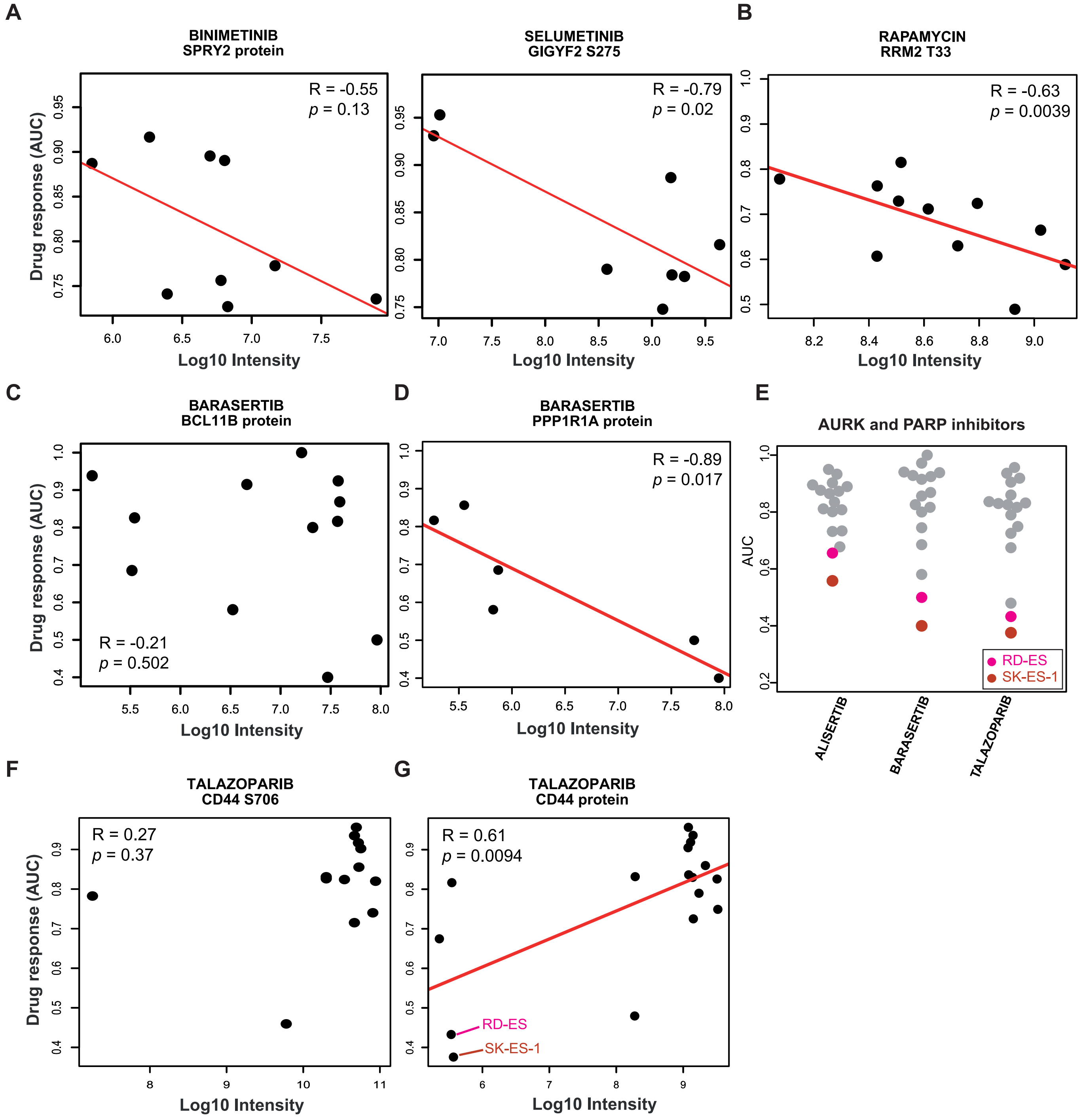

Figure EV4. Examples of correlation analysis of phenotypic drug sensitivity and protein or p-site abundance for drugs forming clusters in sparse multiblock partial least-square regression (SMBPLSR) analysis.

(**A**) SPRY2 protein levels as a marker for sensitivity towards Binimetinib (left panel) and GIGYF2 S275 phosphorylation abundance as a sensitivity marker for Selumetinib (right panel; both drugs are MAP2K inhibitors). (**B–D**) Same as panel (**A**) but for Rapamycin (mTOR inhibitor) and Barasertib (PARP inhibitor). (**E**) The response of two Ewing sarcoma lines (in red and pink) towards AURK and PARP inhibitors is more pronounced than for all other cell lines. (**F, G**) Same as panel A but for Talazoparib (PARP inhibitor). Again, the two Ewing sarcoma lines (in red and pink) are the most sensitive to this drug.

 © The Author(s)

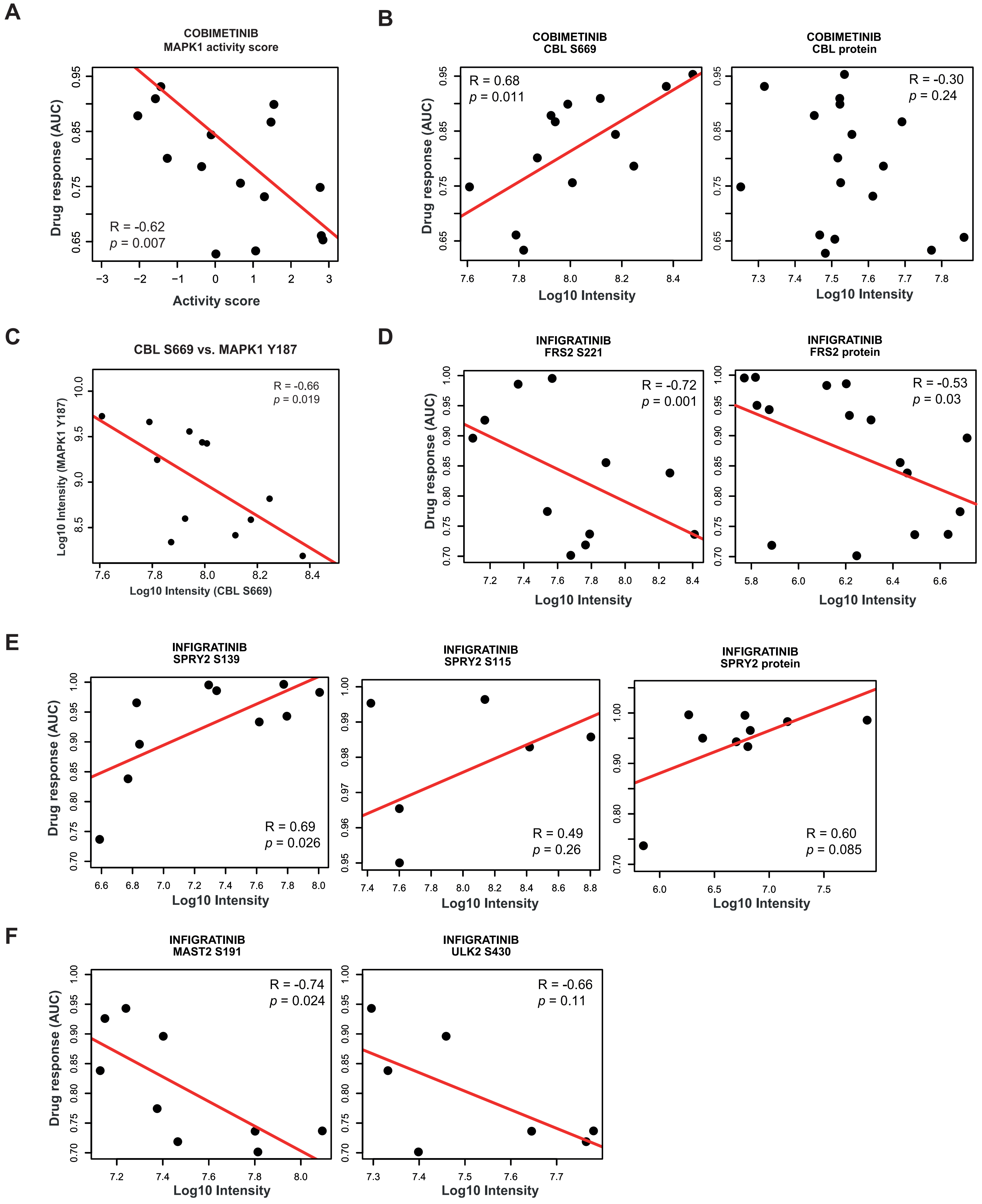
A
COBIMETINIB
MAPK1 activity score
Drug response (AUC)
R = -0.62
p = 0.007
Activity score
B
COBIMETINIB
CBL S669
R = 0.68
p = 0.011
Log10 Intensity
COBIMETINIB
CBL protein
R = -0.30
p = 0.24
Log10 Intensity
C
CBL S669 vs. MAPK1 Y187
Log10 Intensity (MAPK1 Y187)
R = -0.66
p = 0.019
Log10 Intensity (CBL S669)
D
INFIGRATINIB
FRS2 S221
R = -0.72
p = 0.001
Log10 Intensity
INFIGRATINIB
FRS2 protein
R = -0.53
p = 0.03
Log10 Intensity
E
INFIGRATINIB
SPRY2 S139
R = 0.69
p = 0.026
Log10 Intensity
INFIGRATINIB
SPRY2 S115
R = 0.49
p = 0.26
Log10 Intensity
INFIGRATINIB
SPRY2 protein
R = 0.60
p = 0.085
Log10 Intensity
F
INFIGRATINIB
MAST2 S191
R = -0.74
p = 0.024
Log10 Intensity
INFIGRATINIB
ULK2 S430
R = -0.66
p = 0.11
Log10 Intensity

Figure EV5. Examples for correlation analysis of phenotypic drug sensitivity and protein or p-site abundance from candidates identified by elastic net regression.

(**A**) Correlation of MAPK1 activity score and drug responses across sarcoma lines. (**B–F**) Correlation of protein and p-site abundance vs. drug responses across sarcoma lines (**B**, **D–F**). Correlation of the abundance of CBL S669 and MAPK1 Y187 across sarcoma lines (**C**).

 © The Author(s)

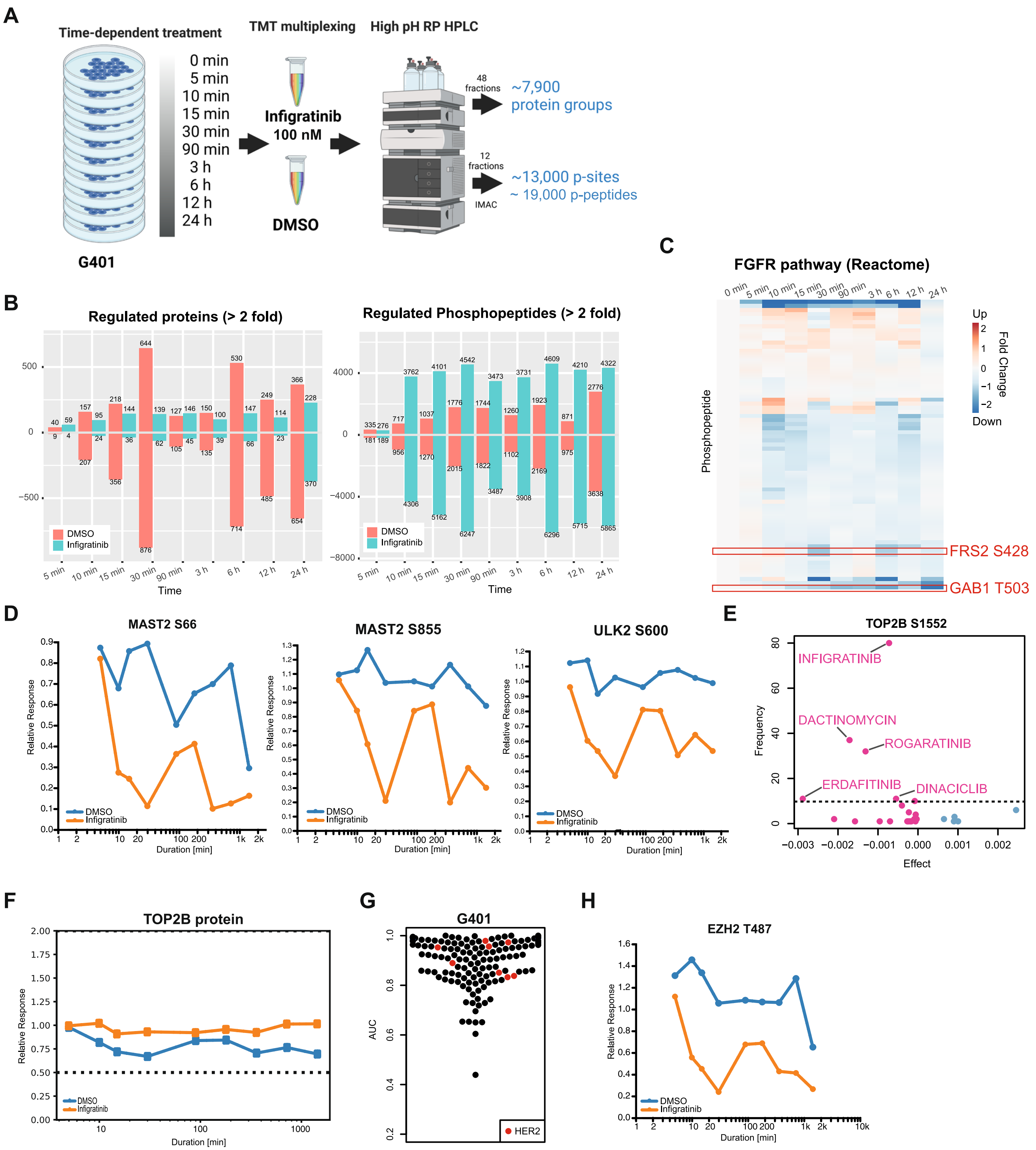
A
Time-dependent treatment
0 min
5 min
10 min
15 min
30 min
90 min
3 h
6 h
12 h
24 h
G401
TMT multiplexing
Infigratinib
100 nM
DMSO
High pH RP HPLC
48 fractions
~7,900 protein groups
12 fractions
IMAC
~13,000 p-sites
~ 19,000 p-peptides
B
Regulated proteins (> 2 fold)
Regulated Phosphopeptides (> 2 fold)
DMSO
Infigratinib
Time
C
FGFR pathway (Reactome)
Phosphopeptide
Up
Down
Fold Change
FRS2 S428
GAB1 T503
D
MAST2 S66
MAST2 S855
ULK2 S600
Relative Response
Duration [min]
E
TOP2B S1552
INFIGRATINIB
DACTINOMYCIN
ROGARATINIB
ERDAFITINIB
DINACICLIB
Frequency
Effect
F
TOP2B protein
G
G401
AUC
HER2
H
EZH2 T487

Figure EV6. Time-resolved (phospho)proteomic analysis of G401 cells in response to Infigratinib.

(**A**) Experimental workflow and number of protein and phosphopeptide identifications/quantification. For each time point of drug treatment, a DMSO control was included to account for abundance changes that are not due to the drug treatment. (**B**) Number of drug-induced protein expression changes (left panel) and phosphopeptide abundance (at least twofold compared to zero min at different time points). (**C**) Heatmap of fold-changes of phosphopeptides from proteins involved in FGFR signalling (annotations from Reactome) compared to DMSO at different time points after Infigratinib treatment. (**D**) Time course of p-sites of MAST2 and ULK2 proteins following Infigratinib treatment. (**E**) Elastic net regression analysis using the phosphorylation site S1552 of TOP2B to identify drugs associated with this p-site. (**F**) TOP2B protein level at different time points after Infigratinib treatment. (**G**) Distribution of AUC values from the phenotypic drug screen in G401 cells (139 kinase inhibitors shown). Inhibitors targeting HER2 are highlighted in red. (**H**) Time course of EZH2 T487 following Infigratinib treatment.

 © The Author(s)
